# Socioeconomic inequality in adolescent life satisfaction: temporal trends and age- and sex-specific patterns from the Norwegian Health Behaviour in School-aged Children surveys, 2002–2018

**DOI:** 10.1186/s12889-026-27695-5

**Published:** 2026-05-13

**Authors:** Martika Irene Brook, Tormod Bøe, Oddrun Samdal, Torill Bogsnes Larsen, Gonneke W J M Stevens, Torbjørn Torsheim

**Affiliations:** 1 Department of Psychosocial Science, Faculty of Psychology, University of Bergen, Bergen, Norway; 2 Regional Centre for Child and Youth Mental Health and Child Welfare, NORCE Norwegian Research Centre, Bergen, Norway; 3 Department of Health Promotion and Development, Faculty of Psychology, University of Bergen, Bergen, Norway; 4 Department of Interdisciplinary Social Science, Faculty of Social and Behavioural Sciences, Utrecht University, Padualaan 14, Utrecht, 3584 CH The Netherlands

**Keywords:** Socioeconomic inequality, Life satisfaction, Adolescents, Family Affluence Scale, HBSC

## Abstract

**Background:**

Life satisfaction is a key outcome indicator of socioeconomic inequality during adolescence. While several countries report socioeconomic inequalities in adolescent life satisfaction, temporal trends and age- and sex-specific patterns remain unclear due to mixed results across national contexts. Inconsistent findings highlight the need for country-specific examinations, particularly in Norway, where research is limited.

**Objectives:**

This study investigates temporal trends and age- and sex-specific patterns of socioeconomic inequality in life satisfaction among Norwegian adolescents.

**Methods:**

Data from five survey cycles of the Norwegian Health Behaviour in School-Aged Children (HBSC) surveys, conducted between 2001/02 and 2017/18, were analysed. Each cycle included 4,000 to 7,000 pupils from all regions of Norway. A regression framework using generalised linear models (GLMs) with a Poisson distribution was employed. Life satisfaction, measured via Cantril’s ladder, served as the dependent variable, while ridit-transformed Family Affluence Scale II (FAS II) served as the independent variable. Age groups, sex, and survey years were incorporated as interaction terms, and the Slope Index of Inequality (SII) and Relative Index of Inequality (RII) were derived from the models.

**Results:**

Adolescents from higher socioeconomic strata reported life satisfaction scores approximately 0.84 points higher (SII) on Cantril’s ladder than their peers in lower strata, with a relative difference of 12% (RII). Temporal trends remained stable. While sex-specific patterns were consistent, socioeconomic inequality increased with age.

**Conclusion:**

Socioeconomic inequality in life satisfaction among Norwegian adolescents persists over time and becomes more pronounced with age, underscoring the need for targeted interventions addressing age-specific inequalities.

## Introduction

Adolescence is a pivotal developmental stage characterised by significant biological and psychosocial changes [1, 2]. During this period, adolescents from socioeconomically disadvantaged backgrounds consistently report poorer health outcomes than their more advantaged peers [3, 4]. As a sensitive developmental window linking childhood conditions with adult outcomes, adolescence represents a critical juncture at which these inequalities may become patterned across the life course, thereby constituting a major public health concern [2, 5].

Systematic monitoring of socioeconomic inequalities in adolescence is essential for informing policies and interventions aimed at promoting health equity [6, 7]. However, because adolescence is characterised by relatively low levels of mortality and serious disease compared with other age groups [8], traditional disease-based indicators are less suited to capturing meaningful variation in health within this population, including socioeconomic gradients. Subjective measures, such as life satisfaction, therefore, offer a more appropriate approach. Life satisfaction assesses adolescents’ overall evaluations of their lives and broader dimensions of well-being [9, 10] and serves as a key indicator of the broader health consequences of socioeconomic inequality during adolescence [2, 11].

The importance of life satisfaction is further underscored by its inclusion in the Organisation of Economic Co-operation and Development (OECD) well-being framework, which tracks societal progress across member countries [12]. Several mechanisms link socioeconomic position (SEP) to adolescent well-being, including parental stress (family stress model), limited social networks (social capital model), and material constraints (family investment model) [13, 14]. A multilevel multidomain approach further underscores the complexity of mechanisms [15].

While absolute and relative socioeconomic inequalities in adolescent life satisfaction have been documented across many countries, their temporal development remains unclear, as findings vary across national contexts [3, 4]. Some studies indicate a decline in socioeconomic inequalities in adolescent life satisfaction over time, whereas others report no clear increase or decrease [11, 16]. This inconsistency suggests that socioeconomic inequalities in adolescent life satisfaction do not follow a universal trajectory, underscoring the need for country-specific analyses.

In this regard, Norway provides a unique socioeconomic context. Despite high living standards, including a gross domestic product (GDP) per capita well above the OECD average, universal healthcare, free education, and generous social benefits [17], socioeconomic inequalities in health persist, a phenomenon referred to as the “Nordic Paradox” [18, 19]. Although adolescent life satisfaction in Norway has generally remained at a relatively high and stable level over time [20, 21], evidence on temporal trends in socioeconomic inequalities in adolescent life satisfaction remains limited. Moreover, little is known about how these inequalities vary across key demographic groups, such as age and sex.

### Demographic variations in socioeconomic inequality

It is important to account for demographic variation, as the association between socioeconomic position and life satisfaction may not be uniform across adolescents. Evidence from international studies indicates that socioeconomic inequalities in adolescent life satisfaction vary by age and sex, although these patterns differ across countries [22]. This heterogeneity underscores the importance of examining how such inequalities are patterned within specific national contexts. In Norway, however, empirical evidence on these demographic differences remains limited, leaving uncertainty as to which groups may be most affected.

Chen et al. [23] proposed three developmental models to explain how SEP-health relationships may evolve from childhood to adolescence. The childhood-adolescent persistent model states that SEP differences appear early and remain constant. The childhood-limited model proposes that SEP effects are initially strong but diminish during adolescence as other factors (e.g., peer relationships) gain importance [24, 25]. The adolescent-emergent model suggests that SEP effects are modest in early childhood but grow stronger during adolescence due to the cumulative impact of disadvantage. Chen et al. [23] emphasised that the model’s relevance depends on the health outcomes under study and on sociocultural and historical contexts. Sex is another important consideration, as socioeconomic inequality in health is often more pronounced among girls than boys (e.g., [26]).

### Methodological challenges in measuring adolescent socioeconomic position

Methodologically, assessing socioeconomic inequalities in adolescence poses unique challenges. Traditional indicators of SEP, such as parental income, often prove unreliable when assessing adolescent populations [27]. The Family Affluence Scale (FAS), widely used in Health Behaviour in School-Aged Children (HBSC) surveys, addresses this issue but has limitations, including changes in measurement properties over time and across demographic groups [28, 29]. To address these concerns, this study follows the methodology of Moreno-Betancur et al. [30], using a regression framework that employs the Slope Index of Inequality (SII) and Relative Index of Inequality (RII) to quantify, in relative and absolute terms, the linear association between affluence rank and life satisfaction within Norway. Based on ranked data, these methods eliminate the need for direct temporal and cross-group comparisons, making them ideal for assessing temporal trends and demographic patterns of socioeconomic inequality in adolescent life satisfaction.

### Aim of the study

Drawing on 16 years of HBSC data from Norway (2001/02 to 2017/18), this study investigates temporal trends and age- and sex-specific patterns in socioeconomic inequality in life satisfaction among Norwegian adolescents. By addressing existing national research gaps and situating the findings within broader international trends, this study provides critical insights into the evolution of life satisfaction inequality during adolescence. The research questions guiding this study are as follows:

- 1a Is there an overall absolute and relative inequality in life satisfaction?
- 1b Is the overall level of inequality different across sex and age groups?
- 2a Is the level of inequality different across time?
- 2b Is the difference in inequality across time a function of sex and age group?

## Method

### Sample

The study utilised data from the Norwegian part of the Health Behaviour in School-aged Children (HBSC) survey, a collaborative cross-national initiative led by the World Health Organisation (WHO). The HBSC survey examines adolescents’ health behaviours, perceptions, well-being, and contextual experiences, following a standardised research protocol [9]. It employs cluster sampling procedures to obtain nationally representative samples of 11-, 13-, and 15-year-olds. In Norway, 16-year-olds have also been included since 1994. Conducted every four years since 1985, this study focuses on data from five survey cycles between 2001/02 and 2017/18. The survey was completed anonymously during school hours, with each cycle including between 4,000 and 7,000 pupils. Participation is voluntary, and informed consent is obtained from caregivers and students before data collection [31–34].

### Dependent variable: life satisfaction

Life satisfaction was measured using Cantril’s Ladder [35], a single-item measure widely used in adolescent population research, including the HBSC survey. Participants rated their current life on an 11-step ladder ranging from 0, indicating the worst possible life, to 10, indicating the best possible life. In adolescent populations, the measure has demonstrated acceptable test–retest reliability and good convergent validity [36]. Higher scores are associated with higher scores on several dimensions of well-being, including self-perception and psychological well-being [37]. The measure also shows overlap with other established well-being measures used in the HBSC, such as the WHO-5 Well-Being Index and the Short Warwick-Edinburgh Mental Wellbeing Scale [38]. Overall, these findings support its use in population-based studies.

### Independent variable: Family Affluence Scale (FAS)

The present study uses FAS version II (FAS II) as it has been computable within HBSC data since 2002. FAS II consists of four questions: “Does your family own a car, van or truck?” Response categories were: No (= 0); Yes, one (= 1); Yes, two or more (= 2). “Do you have your own bedroom for yourself?” Response categories were: No (= 0); Yes (= 1). “During the past 12 months, how many times did you travel away on holiday/vacation with your family?” Response categories were: Not at all (= 0); Once (= 1); Twice (= 2); More than twice (= 3). The 2013/14 survey modified the holiday item to specifically focus on holidays abroad (Torsheim et al. 2016). “How many computers does your family own?” Response categories were: None (= 0); One (= 1); Two (= 2); More than two (= 3) [39].

The ordinal FAS II was transformed into a continuous variable using ridit transformation [40]. The resulting variable is hereafter referred to as ridit FAS. This method assigns scores ranging from 0 (lowest affluence) to 1 (highest affluence), where each value represents the proportion of the population with a lower level of affluence. For individuals at the top of the affluence hierarchy, this proportion approaches 1, while for those at the bottom, it approaches 0. Ridit FAS were referenced within age groups and survey years and subsequently utilised as the primary independent variable in regression models. In these models, the regression coefficient of the ridit FAS provides a direct estimate of the outcome difference between the highest and lowest point of affluence, thereby representing the total inequality within the population [41].

Age, sex, and survey year were applied as covariates and incorporated into interaction terms to assess their influence on socioeconomic inequality in life satisfaction. Age was categorised into four groups: 11-year-olds, 13-year-olds, 15-year-olds, and 16-year-olds. Sex was represented as a categorical variable, where “1” denoted boys and “2” denoted girls. Survey years were classified into five distinct categories: 2002, 2006, 2010, 2014, and 2018.

### Statistical analysis

To assess temporal trends and age- and sex-specific patterns of socioeconomic inequality, the methodology of Moreno-Betancur et al. [30] was followed. A regression framework with generalised linear models (GLMs) using a Poisson distribution was utilised, incorporating interaction effects between ridit FAS and the variables age groups, sex, and survey years.

Design effects from clustered observations within the same class were assessed using the intraclass correlation coefficient (ICC) and design effect (DEFF), stratified by survey year and age category. Clustering was modest (ICC = 0.03; DEFF = 1.56), corresponding to an approximate 56% increase in variance relative to simple random sampling and approximately 25% larger standard errors, indicating reduced statistical precision. However, this level of clustering is unlikely to bias the conclusions of the present analyses; therefore, no adjustment for clustering was applied.

Research question 1a, concerning overall inequality, was tested using a main effects model. Research question 1b (differences in inequality across sex and age groups) and research question 2a (time trends in inequalities across survey years) were assessed using two-way interaction models. Research question 2b (different time trends in inequalities across age groups and sex) was assessed through three-way and four-way interaction models. Poisson GLMs were estimated using maximum likelihood (ML) and were reported with 95% confidence intervals (CIs) [30, 42].

Type III likelihood-ratio tests (LRTs) and deviance statistics were used to evaluate model fit and identify significant interaction terms. Diagnostic checks identified underdispersion (dispersion = 0.47) within the models; therefore, Quasi-Poisson adjustments were applied to control for significant interactions using robust standard errors and CIs. Model pruning was performed based on significant interactions to retain only significant terms, with a significance threshold set at *p* < .05.

The SII and RII were derived from the four-way interaction model using the *emtrends* function from the *emmeans* package [43]. A logarithmic link function was applied to calculate RII, and the resulting coefficients were exponentiated. The SII was calculated using an identity link function. The SII is interpreted as the absolute difference in life satisfaction between the lowest and highest point of affluence (rate differences). For example, an SII of 1 indicates a one-unit difference in life satisfaction, while SII of 0 indicates no absolute difference in life satisfaction. Conversely, the exponentiated RII represents the relative difference in life satisfaction between the lowest and highest levels of affluence (rate ratios). For instance, an RII of 1.10 suggests that life satisfaction at the highest level of affluence is 10% greater than the lowest level, while RII of 1 indicates no relative inequality in life satisfaction. Together, the SII and RII provide a comprehensive view of health inequalities, capturing both the absolute magnitude of inequality (SII) and the proportional differences in inequality (RII) across socioeconomic strata [30]. All data analyses were conducted in R for Windows [44].

## Results

The study analysed data from 29,806 Norwegian adolescents. The overall sample was evenly split between boys (50%) and girls (50%), with age groups distributed as follows: 11-year-olds (28%), 13-year-olds (22%), 15-year-olds (21%) and 16-year-olds (29%). Table 1 provides an overview of the overall sample characteristics by survey year. Life satisfaction scores were relatively stable over time, with mean values varying little between survey years (M = 7.33–7.74), suggesting no substantial changes in average life satisfaction among Norwegian adolescents during the study period.

**Table 1:** Overall sample characteristics by survey year

	Overall (*n* = 29,806)	2002 (*n* = 7,031)	2006 (*n* = 6,426)	2010 (*n* = 5,756)	2014 (*n* = 4,556)	2018 (*n* = 6,037)
Age						
11 years, n (%)	8,227 (28%)	1,657 (24%)	1,578 (25%)	1,679 (29%)	1,370 (30%)	1,943 (32%)
13 years, n (%)	6,699 (22%)	1,736 (25%)	1,585 (25%)	1,320 (23%)	1,046 (23%)	1,012 (17%)
15 years, n (%)	6,251 (21%)	1,622 (23%)	1,534 (24%)	1,339 (23%)	878 (19%)	878 (15%)
16 years, n (%)	8,629 (29%)	2,016 (29%)	1,729 (27%)	1,418 (25%)	1,262 (28%)	2,204 (37%)
Sex						
Boy, n (%)	14,945 (50%)	3,645 (52%)	3,302 (51%)	2,891 (50%)	2,156 (47%)	2,951 (49%)
Girl, n (%)	14,841 (50%)	3,386 (48%)	3,124 (49%)	2,865 (50%)	2,400 (53%)	3,066 (51%)
Life satisfaction (*M* (*SD*))	7.60 (1.90)	7.33 (1.96)	7.71 (1.86)	7.64 (1.91)	7.74 (1.81)	7.64 (1.91)
Ridit FAS (*M* (*SD*))	0.50 (0.28)	0.50 (0.28)	0.50 (0.28)	0.50 (0.28)	0.50 (0.28)	0.50 (0.28)

Note: Table 1 presents n (%) for age and sex, as well as means (M) with standard deviation (SD) for life satisfaction, and ridit FAS. The ridit FAS were 0.50 (SD = 0.28) across all years due to the transformation applied

### Temporal trends and age- and sex-specific patterns

**Fig. 1:**
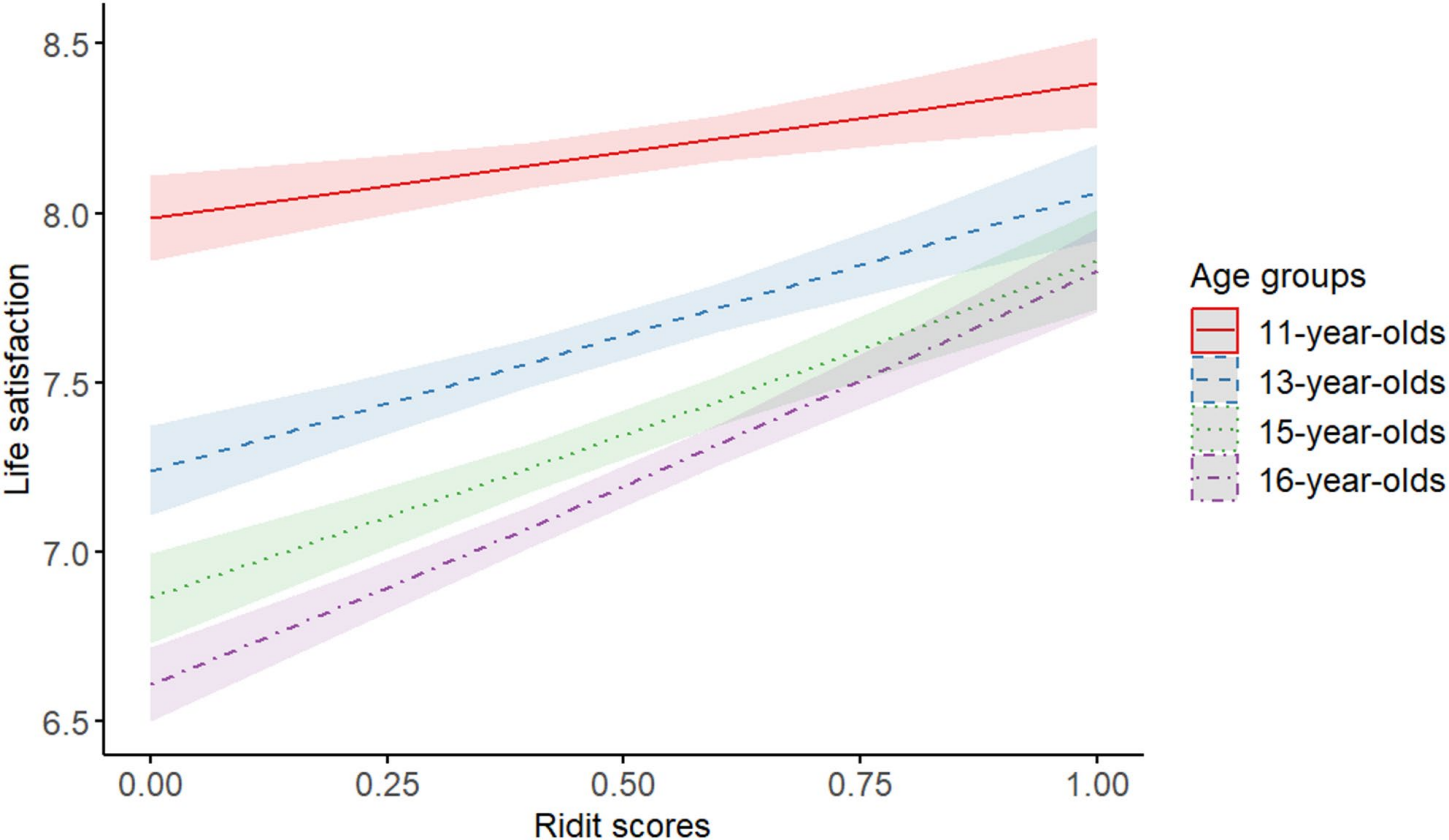
Interaction effects between ridit FAS and age groups on predicted life satisfaction Note: Interaction effect between ridit FAS and age groups on life satisfaction, with lines representing the predicted life satisfaction scores across ridit FAS for different age groups: 11-year-olds (red), 13-year-olds (blue), 15-year-olds (green), and 16-year-olds (purple). The shaded areas around each line denote the 95% CIs

The main effects model provided results relevant to research question 1a, revealing a strong main effect of ridit FAS, suggesting overall inequalities in life satisfaction (χ^2^(1) = 216.71, *p* < .001). Relevant for research question 1b, the two-way interaction model revealed a statistically significant interaction between ridit FAS and age groups (χ^2^(3) = 35.87, *p* < .001), indicating age group differences in socioeconomic inequalities in life satisfaction, as illustrated in Fig. 1. The interaction between ridit FAS and sex was non-significant (χ^2^(1) = 0.19, *p* = .663), suggesting no evidence of sex differences in inequalities. As a test of research question 2a and b, the interaction between ridit FAS and survey year was statistically non-significant (χ^2^(4) = 4.07, *p* = .396), indicating a lack of evidence for time trends in inequalities. The three-way and four-way interaction models did not achieve significance, indicating no evidence of temporal trends as a function of sex and age group.

In addition to the interaction effects that were focal to the research questions, the SII and RII were derived from the four-way interaction model to further assess the absolute and relative effects of socioeconomic inequality, relevant to research question 1a and 1b. Table 2 presents the results of the analysis of socioeconomic inequalities in life satisfaction across survey years, age groups and sex in the Norwegian HBSC survey, expressed as SII and rate ratios (exponentiated RII), with accompanying 95% CIs.

**Table 2:** Socioeconomic inequality in life satisfaction across time, sex and age groups

Demographic	2002 Est. (95% CI)	2006 Est. (95% CI)	2010 Est. (95% CI)	2014 Est. (95% CI)	2018 Est. (95% CI)	Overall Est. (95% CI)
Overall						
RR	1.13 (1.09–1.16)	1.09 (1.06–1.13)	1.15 (1.11–1.19)	1.11 (1.07–1.16)	1.11 (1.07–1.15)	1.12 (1.10–1.14)
SII	0.86 (0.63–1.08)	0.69 (0.44–0.93)	1.03 (0.78–1.29)	0.82 (0.52–1.13)	0.80 (0.53–1.08)	0.84 (0.72–0.96)
Boys						
RR	1.11 (1.07–1.16)	1.10 (1.05–1.15)	1.13 (1.08–1.19)	1.13 (1.07–1.20)	1.10 (1.04–1.15)	1.11 (1.09–1.14)
SII	0.79 (0.47–1.11)	0.76 (0.41–1.11)	0.98 (0.61–1.35)	0.97 (0.52–1.42)	0.71 (0.31–1.10)	0.84 (0.67–1.01)
Girls						
RR	1.14 (1.09–1.19)	1.09 (1.04–1.14)	1.16 (1.11–1.22)	1.10 (1.04–1.16)	1.13 (1.07–1.19)	1.12 (1.10–1.15)
SII	0.92 (0.60–1.25)	0.61 (0.26–0.97)	1.09 (0.73–1.45)	0.68 (0.28–1.08)	0.90 (0.52–1.28)	0.84 (0.68-1.00)
11-year-olds						
RR	1.04 (0.98–1.10)	1.04 (0.98–1.11)	1.05 (0.99–1.11)	1.08 (1.01–1.16)	1.05 (0.99–1.11)	1.05 (1.02–1.08)
SII	0.29 (-0.20–0.78)	0.32 (-0.21–0.84)	0.40 (-0.10–0.90)	0.66 (0.10–1.23)	0.40 (-0.07–0.87)	0.41 (0.19–0.64)
13-year-olds						
RR	1.13 (1.07–1.21)	1.08 (1.02–1.16)	1.15 (1.07–1.24)	1.12 (1.03–1.21)	1.07 (0.98–1.16)	1.11 (1.08–1.15)
SII	0.94 (0.48–1.40)	0.66 (0.15–1.16)	1.09 (0.55–1.63)	0.86 (0.25–1.48)	0.48 (-0.14–1.09)	0.81 (0.56–1.05)
15-year-olds						
RR	1.16 (1.09–1.24)	1.14 (1.06–1.21)	1.18 (1.10–1.27)	1.11 (1.02–1.22)	1.10 (1.01–1.20)	1.14 (1.10–1.78)
SII	1.08 (0.62–1.54)	0.95 (0.46–1.44)	1.23 (0.70–1.75)	0.81 (0.13–1.50)	0.72 (0.06–1.38)	0.96 (0.70–1.21)
16-year-olds						
RR	1.17 (1.11–1.24)	1.12 (1.05–1.19)	1.22 (1.14–1.31)	1.14 (1.06–1.23)	1.24 (1.17–1.32)	1.18 (1.14–1.21)
SII	1.11 (0.70–1.52)	0.82 (0.37–1.28)	1.42 (0.92–1.91)	0.95 (0.40–1.50)	1.62 (1.21–2.02)	1.18 (0.98–1.39)

Note: Coefficients β are calculated using generalised linear models. For the SII, an identity link function is used to indicate absolute differences. A log link function is used for the RII, and the resulting coefficients are exponentiated to represent Rate Ratios. 95% CIs are presented in brackets

Socioeconomic inequalities in life satisfaction were evident across all subgroups. Overall, individuals at the highest end of the socioeconomic distribution (ridit FAS = 1) reported life satisfaction scores approximately 0.84 points higher (SII) than those at the lowest end (ridit FAS = 0), with a proportional difference of 1.12 (rate ratios). The latter suggests that life satisfaction is about 12% higher for adolescents at the top of the socioeconomic distribution than for those at the bottom. For girls, the overall effect was 0.84 (SII) with a proportional difference of 1.12. Boys showed a similar overall effect. Among 16-year-olds, the overall effect was 1.18 (SII) with a proportional difference of 1.18, indicating stronger effect than 11-year-olds, who had an overall effect of 0.41 (SII) with a proportional difference of 1.05.

**Fig. 2:**
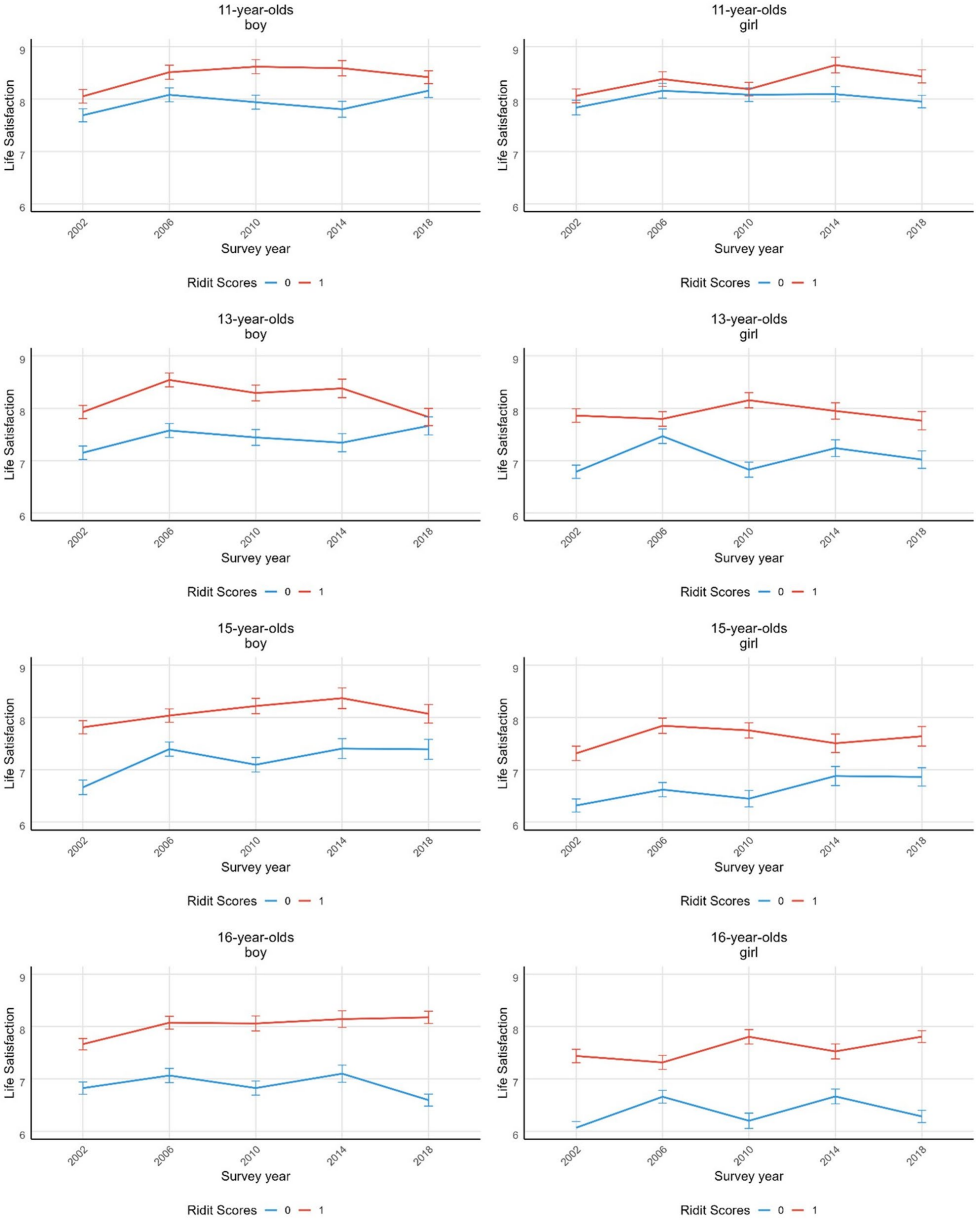
Trends in adolescent life satisfaction across socioeconomic strata Note: The panels show predicted life satisfaction across survey years, stratified by sex (boys and girls) and age group (11-, 13-, 15-, and 16-year-olds). Life satisfaction is compared at the lowest (ridit FAS = 0) and highest (ridit FAS = 1) positions in the socioeconomic distribution. Error bars indicate 95% CIs

Figure 2 visualises predicted life satisfaction across survey years, with separate panels by age group (11-, 13-, 15-, and 16-year-olds) and sex (boys and girls), at the lowest (ridit FAS = 0) and highest (ridit FAS = 1) levels of affluence. The difference in life satisfaction between the highest and lowest ridit FAS is relatively stable and significant across panels. The key observation is the widening gap in life satisfaction scores between the lower and upper socioeconomic standing as age increases, highlighting the main finding of the study: socioeconomic inequality in life satisfaction grows with age. For 16-year-olds, the difference in life satisfaction between the highest and lowest affluence is notably stronger than for 11-year-olds.

## Discussion

This study examined socioeconomic inequalities in life satisfaction among Norwegian adolescents by analysing HBSC data from 29,806 participants over a 16-year period (2001/02–2017/18). The findings revealed both absolute and relative socioeconomic inequalities in life satisfaction. Temporal trends remained stable, with no significant changes observed over time. While sex-specific patterns were consistent, socioeconomic inequality increased with age.

### Temporal stability of trends

Findings revealed both absolute (SII) and relative (RII) socioeconomic inequality in adolescent life satisfaction. This finding is consistent with prior research linking higher SEP to higher life satisfaction in adolescents [45, 46]. Over a 16-year span in Norway, the present study observed a consistent pattern of socioeconomic inequality in adolescent life satisfaction, with no significant trend toward temporal increase or decrease.

Research from other HBSC countries has produced mixed findings. While some studies report no clear increase or decrease over time, others indicate slight declines (e.g., [11, 47]). Although the reasons for mixed findings across countries are not fully understood, they may reflect that socioeconomic inequalities in adolescent life satisfaction are shaped through multiple pathways, and the salience of these pathways may vary across countries and time depending on broader economic and social contexts [15, 46]. Methodological differences may also be relevant; for instance, different indicators of socioeconomic position may capture distinct dimensions of inequalities over time [48, 49].

Although rising income inequality has been linked to widening socioeconomic inequalities in life satisfaction (e.g., [3, 46]), Norway presents an intriguing contrast. Despite the country’s rising Gini coefficient over recent decades [50], this study found no evidence of widening socioeconomic inequality in adolescent life satisfaction over time.

Mean levels of adolescent life satisfaction were relatively high and stable across survey years, consistent with previous Norwegian studies [20, 21]. The coexistence of stable average levels of life satisfaction and persistent socioeconomic inequalities suggests that neither overall life satisfaction nor inequality has substantially changed during the study period. One possible interpretation is that Norway’s comparatively egalitarian welfare state and longstanding policy commitment to reducing socioeconomic inequalities may buffer adolescents against the effects of rising income inequality [51].

At the same time, the persistence of socioeconomic inequalities in adolescent life satisfaction indicates that such policies may be insufficient to reduce inequality itself, even if they help prevent its further widening. Given that reducing socioeconomic inequalities in health has been a central objective of Norwegian public health policy [51], the present findings suggest that this goal has not yet been fully realised with regard to adolescent life satisfaction.

### Age- and sex-specific patterns

As adolescents grow older, they appear to be increasingly impacted by their SEP, with 16-year-olds being more affected than 11-year-olds. Although patterns vary across countries, similar age-related patterns have been reported in other HBSC countries [22]. Chen et al. [23] emphasised the importance of sociocultural context in interpreting such patterns, and variation in age-specific inequalities observed across countries may suggest that there is no universal development trajectory. In Norway, however, the present findings indicate that late adolescence may be a particularly important period for socioeconomic inequality in life satisfaction.

The widening socioeconomic inequality with age is consistent with the adolescent-emergent model proposed by Chen et al. [23], which posits that socioeconomic inequality intensifies during adolescence. While several complex mechanisms link SEP to adolescent well-being [13, 14], an adolescent-emergent pattern underscores the importance of considering mediators in the SEP-life satisfaction relationship that may become more prominent during adolescence. For instance, increased social comparisons. Multiple discrepancy theory (MDT) [52] provides a useful framework in this regard, positing that life satisfaction is shaped by perceived discrepancies between one’s current circumstances and multiple reference standards, such as what one wants, needs, deserves, or perceives others to have. In the context of socioeconomic inequality, adolescents from lower socioeconomic backgrounds may be more likely to experience unfavourable comparisons with more advantaged peers.

Van der Aar et al. [53] demonstrated that mid-adolescents (15–17 years) are particularly sensitive to the negative effects of social comparisons. Heightened sensitivity to social evaluation may amplify their awareness of socioeconomic differences within peer groups, especially regarding visible markers of affluence, such as access to technology and opportunities for leisure activities. Moreover, as adolescents grow older, the scope of social comparisons may expand to include broader contexts beyond direct peers and immediate environments, such as online platforms. These broader comparisons may increase unfavourable self-evaluations among adolescents from lower socioeconomic backgrounds, potentially exacerbating socioeconomic inequality in life satisfaction.

The present study did not investigate the mediating mechanisms underlying age-related trends. It would be interesting for future research to investigate the interplay between developmental processes, social comparisons, and the role of family affluence, as this may provide valuable insights into factors shaping life satisfaction during adolescence.

Sex-specific patterns remained stable, indicating that the magnitude of socioeconomic inequality in life satisfaction did not significantly differ between boys and girls in the Norwegian context. Previous research has noted varying sex-specific patterns of socioeconomic inequalities in life satisfaction based on national contexts [22]. The reason for variation in sex-specific patterns is unclear but could potentially reflect contextual factors such as gender role tradition or cultural expectation [22, 54]. The present results indicate that in Norway, sex may not be a significant moderating factor in the relationship between SEP and life satisfaction among adolescents.

### Methodological considerations and limitations

Several methodological considerations warrant attention. First, the observed stability in life satisfaction inequality over time may partly reflect limitations in the tool used to measure family affluence. FAS II has been found to lose discriminatory power in affluent countries, where a large proportion of participants cluster in the highest FAS categories [27, 39]. This limitation is particularly relevant in Norway, where high living standards and a wealthy population may prevent FAS II from capturing meaningful variations among the most affluent. Consequently, the observed stability in socioeconomic inequality may partly reflect the scale’s lower ability to detect rising inequalities at the upper end of the affluence spectrum.

Additionally, Norwegian schools have undergone significant digital transformation, with one-to-one provision introduced in upper secondary education between 2007 and 2009 and later expanded unevenly across compulsory education, particularly from around 2016 onwards [55, 56]. As this rollout occurred unevenly across educational stages, it may have influenced both temporal and age-related patterns in the present analyses. In particular, access to computers increasingly reflects not only household material resources but also publicly provided infrastructure. This likely reduced the discriminatory capacity of the computer item within the FAS, especially at the lower end of the socioeconomic distribution, as access to at least one device became more widespread over time and earlier among older students.

Consequently, distinctions between lower and middle socioeconomic groups may have become smaller over time, potentially attenuating socioeconomic gradients in life satisfaction. However, given the composite nature of the FAS and continued differentiation at higher levels of material resources, for example, access to multiple devices, this effect is unlikely to account for the observed persistence of inequalities over time or their increase with age. If anything, these estimates may be conservative, and both age-related differences and temporal trends should be interpreted with this potential underestimation in mind.

The development of FAS III aimed to address the limitations of reduced discriminatory power by incorporating more contemporary indicators of family affluence [57]. While FAS III may improve the scale’s discriminatory power, further refinements, such as adding indicators of higher affluence, including luxury goods, could enhance its applicability in wealthy contexts like Norway by better capturing variations at the upper levels of the affluence spectrum.

Second, measurement non-invariance is a limitation inherent to the FAS II measure rather than the study itself. Measurement non-invariance means that the dimensions underpinning the measure of family affluence are not directly comparable across age, sex, or time [27]. The current study referenced ridit FAS within separate groups and employed SII and RII to eliminate the need for direct temporal or cross-group comparisons. Future research could benefit from developing more invariant indicators to enhance the robustness of analyses.

Third, this study did not examine mediating mechanisms linking family affluence to adolescent life satisfaction. The family stress model, family investment model, and social capital perspectives highlight key pathways through which socioeconomic conditions may shape life satisfaction, including parental stress, parenting practices, material and educational resources, and supportive social relationships [13, 14]. Future research could incorporate such theoretically grounded mediators to better understand the mechanisms underlying these associations.

## Conclusions

This study highlights persistent socioeconomic inequalities in life satisfaction among Norwegian adolescents, which were stable over time and consistent across sexes. A notable finding is the increase in socioeconomic inequality with age, irrespective of historical time. These findings underscore the need for targeted interventions addressing age-specific socioeconomic inequalities in adolescent life satisfaction.

## Data Availability

Data are available from the HBSC Institutional Data access (https://www.uib.no/en/hbscdata) for external researchers who meet the criteria for access to the data.

## Abbreviations

CI: confidence interval
DEFF: design effect
FAS: Family Affluence Scale
FAS II: Family Affluence Scale II
GDP: gross domestic product
GLM: generalised linear model
HBSC: Health Behaviour in School-aged Children
ICC: intraclass correlation coefficient
LRT: likelihood-ratio test
M: mean
MDT: Multiple Discrepancy Theory
ML: maximum likelihood
OECD: Organisation for Economic Co-operation and Development
RII: Relative Index of Inequality
SD: standard deviation
SEP: socioeconomic position
SII: Slope Index of Inequality
WHO: World Health Organisation
